# Right lower quadrant abdominal mass: A case report

**DOI:** 10.1002/ccr3.6544

**Published:** 2022-11-06

**Authors:** Anup Chalise, Lok Bahadur Kathayat, Prabhat Poudel, Ashish Prasad Rajbhandari, Rabin Koirala

**Affiliations:** ^1^ Nepal Medical College and Teaching Hospital Kathmandu Nepal

**Keywords:** emergency surgery, gastric ulcer, gastrointestinal complications, hollow viscus perforation, perforation peritonitis

## Abstract

Right lower quadrant mass in the abdomen has many causes, but gastric perforation is rare. We discuss a 65‐year‐old lady who presented with a history of pain in her abdomen followed by swelling in her right lower abdomen. During the evaluation, a diagnosis of gastric perforation was made.

## Abstract 2

Although late presentations often lead to rapid deterioration with fatal outcomes, proceeding with a step‐wise approach is helpful. Common diagnoses may mislead the physician while the underlying condition remains undiagnosed.

## 1 INTRODUCTION

Gastric perforation is a full‐thickness injury of the stomach wall that communicates between the gastric lumen and the peritoneal cavity.^1^ In acute perforations, the luminal contents freely enter the general peritoneal cavity, causing chemical peritonitis, while the perforations occurring over a prolonged period may be contained locally by an inflammatory reaction resulting in the formation of an abscess.^2^ Although there have been no reports of abscess from gastric perforation tracking down the abdomen peritoneally, retroperitoneally, or even subcutaneously to form an abscess in the right iliac fossa, it is anatomically feasible. Here, we present a case of chronic gastric perforation that presented as a swelling over the right lower abdomen. Although many reports of chronic gastric ulcers are present on a PubMed search, reports of chronic perforations are infrequent.

## 2 PATIENT INFORMATION

A 65‐year‐old lady presented to our surgical clinic with a history of sharp, pricking pain in the epigastrium 1 month ago; after this, she noted a gradually increasing swelling in the right side of the abdomen. She denied a history of severe pain limiting daily activities, fever, shortness of breath, nausea, vomiting, hematemesis, per rectal bleed, loss of appetite, or loss of weight. She had no diagnosed comorbidities till the date of presentation. She was a regular alcohol consumer (local homemade alcohol, two glasses a day) but left a month before the onset of symptoms. She smokes cigarettes and has a 20‐pack‐year history. There is no family history of malignancy or similar illnesses. She had been moving her bowels and passing urine before the hospital visit.

**FIGURE 1:**
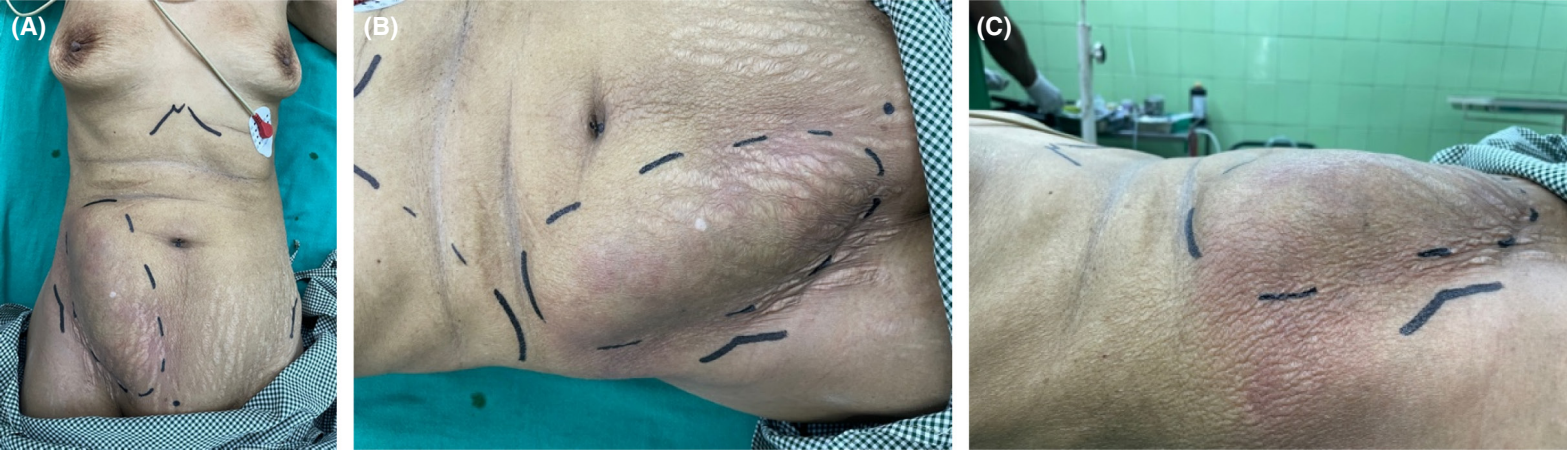
(A) Top view of patients lump (dotted marking); (B) close‐up view of the same lump; (C) lateral view of same lump

On initial examination, the patient was ill‐looking, tachycardic (heart rate 110 bpm), hypotensive (blood pressure 80/60 mmHg), afebrile, looked pale, and had bilateral pedal pitting edema. Abdomen examination revealed a lump (see Figure 1) in the right iliac fossa, ~15 cm × 7 cm in size, extending up to the right hypochondrium with erythema around the overlying skin, tenderness, guarding, but no rigidity. An initial diagnosis of appendicular perforation was made, and the patient worked up.

**FIGURE 2:**
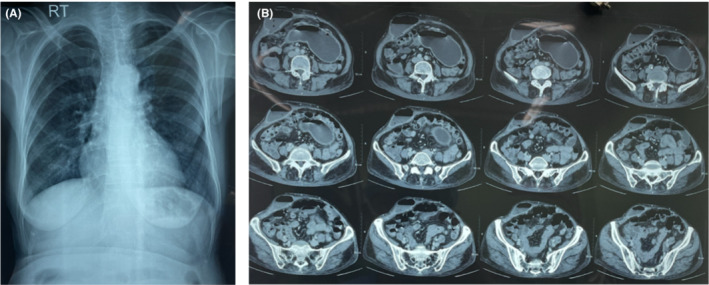
(A) Chest X‐ray without evidence of pneumoperitoneum; (B) CT scan showing a subcutaneous (54 mm × 28 mm) right anterior abdominal wall collection with air fluid level in continuity with a collection within the abdominal cavity (87 mm × 12 mm), further continuous with another collection near the left border of the stomach (90 mm × 23 mm) with suspicious breach of the pylorus of the stomach.

The patient was resuscitated, stabilized, and initial investigations showed a total count of 12,900/mm,^3^ with 80% neutrophils, a hemoglobin of 8.6 g/dl, erythrocyte sedimentation rate of 28 mm in the 1st hour, normal renal function, no pneumoperitoneum on an initial chest X‐ray, and, so, a computed tomography (CT) scan was performed (see Figure 2). Her blood gas and lactate were within normal limits, but her serum albumin was 2.8 g/dl.

**FIGURE 3:**
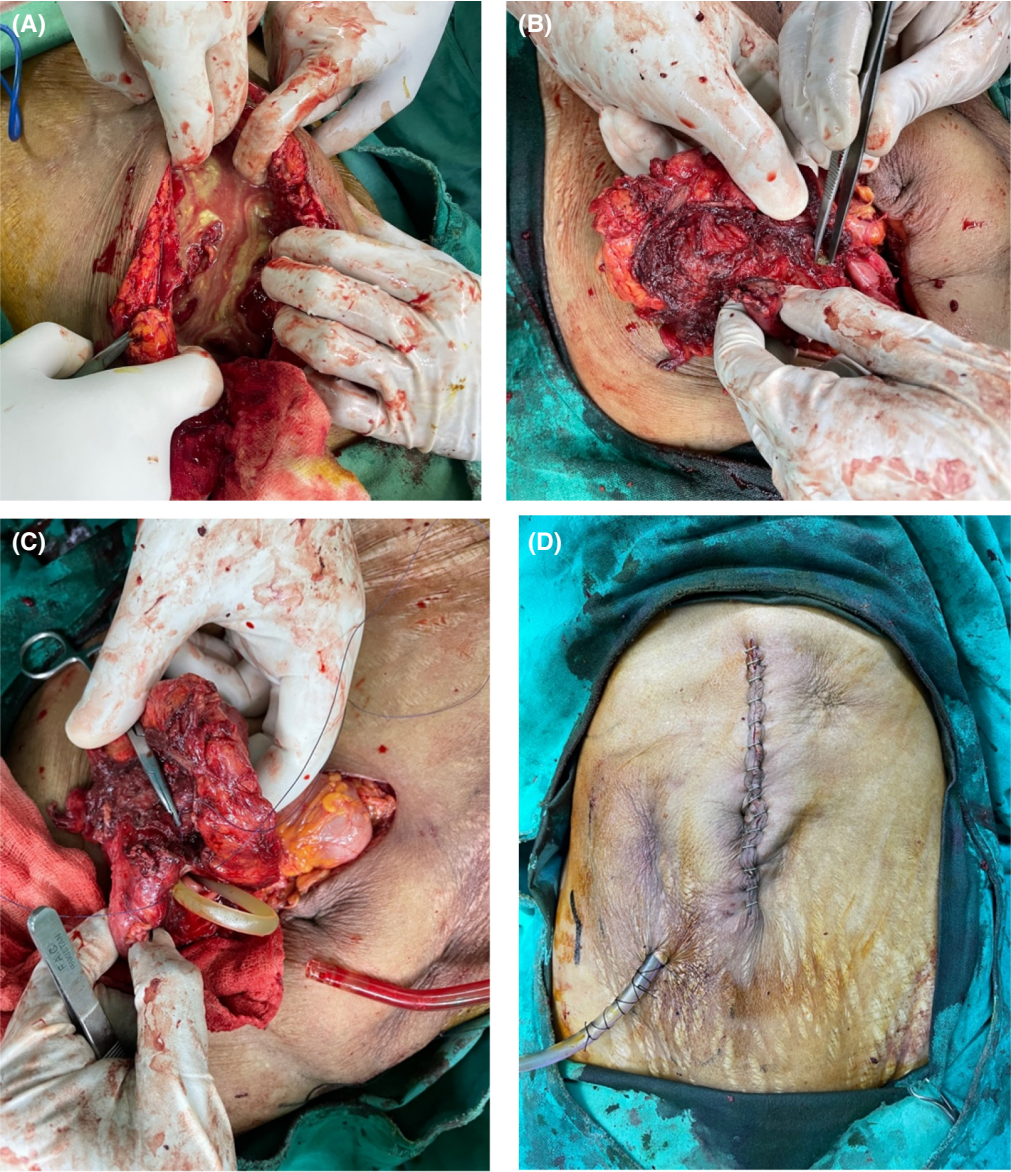
From left, (A) pus collection in the intraabdominal cavity; (B) site of perforation shown by forceps; (C) repair of the perforation; (D) post surgery abdominal incision closure with drain placement

The patient and her caretaker were given the options of percutaneous drainage and surgery. They agreed to the latter. After resuscitation, the patient's vitals had normalized, so the patient underwent exploratory laparotomy via a midline incision on the same day of presentation at our center (see Figure 3). A tract extended from the intraabdominal collection to the right iliac fossa, with a well‐loculated collection surrounding the greater curvature and pylorus of the stomach. Pus was aspirated, sent for culture and sensitivity, suctioned, lavaged, and the abdomen explored. A 5 mm × 5 mm perforation was seen at the greater curvature, approximately 3 cm from the pylorus encased by the fundus of the gallbladder, D1 portion of the duodenum, body of the stomach, inferior border of the right lobe of the liver (right to the falciform ligament), and sealed above by the omentum. On‐table endoscopy failed to identify any mass, so a wedge biopsy with a primary repair with an omental patch was done, and the biopsy specimen was sent for histopathology. The gallbladder perforated and tore down from the undersurface of the liver during dissection, so a cholecystectomy was done. A nasogastric tube was placed in free drain, and a drain was left in the subhepatic pouch. The patient steadily recovered, acquiring only a superficial surgical site infection with total parenteral nutrition support.

Pus culture from the intraabdominal collection was sterile, while the biopsy showed no features suggestive of malignancy. However, *Helicobacter pylori* was identified in the biopsy specimen. The drain was removed on post‐op day seven once it drained <10 ml/24 h. The patient was discharged on post‐op day eight on triple therapy regimen and was followed up in the clinic for regular dressings, secondary suturing, and stitches removal. In her follow‐up 2 months post‐surgery, the patient was doing well and had significantly recovered.

## 3 DISCUSSION

Peptic ulcer disease has four significant complications: bleeding, perforation, penetration, and obstruction. Most gastric perforations occur secondary to peptic ulcer disease, but they can also follow malignancy (~30%), infection, external trauma, ingestion of sharp foreign bodies, interventional procedures, and ischemia.^3^, ^4^ The lifetime risk of benign gastroduodenal perforation is 10% in untreated PUD, and 30%–50% of ulcer perforations are associated with NSAIDs.^5^, ^6^ The high prevalence of *H. pylori* in the low socioeconomic classes with poverty and poor hygiene may have increased the incidence of gastric perforations in the developing world.^7^

The clinical presentation of upper gastrointestinal perforation is a classic triad of sudden onset of abdominal pain, tachycardia, and abdominal rigidity. However, the presentation of gastric perforation may range from mild localized pain to signs of peritonitis and shock depending upon the location and size of the perforation, the amount of leak, and the nature of the body's reaction to the leakage.^4^ However, patients with chronic gastrointestinal perforation can present with vague abdominal symptoms and nonspecific imaging findings, which impede timely diagnosis, similar to our case.

The diagnosis of gastrointestinal perforation is made by identifying extraluminal leakage of gas identified in a plain film chest or erect abdominal X‐ray.^1^ However, our case did not have such a radiological finding. In cases of chronic perforation, ambiguous clinical history without the abrupt onset of abdominal pain and absence of peritoneal irritation makes early diagnosis of perforation difficult. The formation of abscesses around the stomach or in the abdominal wall as a result of the sealed perforation can be detected by USG as an echo‐poor mass with air shadows, fluid densities within the cavity, and irregular borders.^1^ Perforations can also be localized by endoscopy, as in our case. CT scan of the abdomen with and without contrast can also be a valuable tool in locating the perforation, abscess, and sinus tracts. Moreover, CT can be beneficial in predicting the etiology of the perforation and planning its management.^8^ We were able to diagnose the patient early on during the presentation due to the timely availability of a CT scan, which is still not available in many facilities in our country.

Most patients undergo management following the SNAP protocol, which includes management of sepsis, followed by nutrition enhancement, delineation of anatomy, and a definite procedure.^9^ Although this primarily applies to fistulas, chronic cases are usually managed similarly. Since the patient had an abscess, and the management of sepsis requires source control, we had to drain it. Percutaneous drainage was considered, but a laparotomy was performed since the patient, and her caretakers opted for surgical drainage.

Management is mainly surgical, with the repair of the defect, drainage of the abscess, and excision of the infected foci aided by medical management with decompression of the gastrointestinal tract, antibiotic coverage, and bowel rest to allow for healing. Surgery can be done by laparotomy, laparoscopically or by endoscopic closure (i.e., NOTES).^4^, ^10^, ^11^, ^12^ The repair can be a primary or modified Graham's patch repair. It may involve the excision of a wedge of unhealthy tissue and, deemed necessary, can involve gastric body partition or a subtotal gastrectomy followed by reconstruction of the gastrointestinal tract.^1^, ^10^ We chose an open repair due to the patient's status, chronicity of the condition, and for performing a quick damage control surgery, if needed, based on the patient's consent.

Although a late presentation may be commonly encountered, patients often deteriorate rapidly with fatal outcomes. Sometimes common diagnoses considered in such scenarios may mislead the physician when the underlying condition may remain undiagnosed. Thus, we can learn from this case that it is imperative to try and delineate the anatomy of the patient's issues prior to proceeding with surgery right away.

## 4 PATIENT PERSPECTIVE

The patient and attendants believe that if adequate services were available in their rural hospitals, they would have sought help much earlier.

## 5 CONCLUSION

This patient's presentation was infrequently cited in PubMed‐indexed literature, so we reported it. Thus, we can learn that in places like ours in Nepal, where healthcare services and resources do not meet people's needs, such cases can still be treated successfully if timely identification of the underlying disorder can be carried out.

## AUTHOR CONTRIBUTIONS

AC directly handled the case and were directly involved in managing the patient pre‐operatively, intraoperatively, and post‐operatively. They were all involved in planning, writing, and reviewing the case. LBK directly handled the case and were directly involved in managing the patient pre‐operatively, intraoperatively, and post‐operatively. They were all involved in planning, writing, and reviewing the case. PP directly handled the case and were directly involved in managing the patient pre‐operatively, intraoperatively, and post‐operatively. They were all involved in planning, writing, and reviewing the case. APR directly handled the case and were directly involved in managing the patient pre‐operatively, intraoperatively, and post‐operatively. They were all involved in planning, writing, and reviewing the case. RK was directly involved in supervising the case management, conducting patient rounds to ensure patient well‐being, and was also involved in planning, writing, and reviewing the case.

## FUNDING INFORMATION

None.

## CONFLICT OF INTEREST

None.

## CONSENT

Written informed consent was obtained from the patient to publish this report in accordance with the journal's patient consent policy. Original copy has been placed on patient's file.

## Data Availability

Data sharing not applicable to this article as no datasets were generated or analysed during the current study.
